# Identification of an epicuticular wax crystal deficiency gene *Brwdm1* in Chinese cabbage (*Brassica campestris* L. ssp. *pekinensis*)

**DOI:** 10.3389/fpls.2023.1161181

**Published:** 2023-05-30

**Authors:** Gengxing Song, Chuanhong Liu, Bing Fang, Jie Ren, Hui Feng

**Affiliations:** College of Horticulture, Shenyang Agricultural University, Shenyang, China

**Keywords:** Chinese cabbage, EMS mutagenesis, epicuticular wax crystals deficiency, allelic mutants, primary alcohols

## Abstract

**Introduction:**

The cuticle wax covering the plant surface is a whitish hydrophobic protective barrier in Chinese cabbage, and the epicuticular wax crystal deficiency normally has higher commodity value for a tender texture and glossy appearance. Herein, two allelic epicuticular wax crystal deficiency mutants, *wdm1* and *wdm7*, were obtained from the EMS mutagenesis population of a Chinese cabbage DH line ‘FT’.

**Methods:**

The cuticle wax morphology was observed by Cryo-scanning electron microscopy (Cryo-SEM) and the composition of wax was determined by GC-MS. The candidate mutant gene was found by MutMap and validated by KASP. The function of candidate gene was verified by allelic variation.

**Results:**

The mutants had fewer wax crystals and lower leaf primary alcohol and ester content. Genetic analysis revealed that the epicuticular wax crystal deficiency phenotype was controlled by a recessive nuclear gene, named Brwdm1. MutMap and KASP analyses indicated that *BraA01g004350.3C*, encoding an alcohol-forming fatty acyl-CoA reductase, was the candidate gene for *Brwdm1*. A SNP 2,113,772 (C to T) variation in the 6^th^ exon of *Brwdm1* in *wdm1* led to the 262^nd^ amino acid substitution from threonine (T) to isoleucine (I), which existed in a rather conserved site among the amino acid sequences from Brwdm1 and its homologs. Meanwhile, the substitution changed the three-dimensional structure of Brwdm1. The SNP 2,114,994 (G to A) in the 10^th^ exon of *Brwdm1* in *wdm7* resulted in the change of the 434^th^ amino acid from valine (V) to isoleucine (I), which occurred in the STERILE domain. KASP genotyping showed that SNP 2,114,994 was co-segregated with glossy phenotype. Compared with the wild type, the relative expression of Brwdm1 was significantly decreased in the leaves, flowers, buds and siliques of wdm1.

**Discussion:**

These results indicated that *Brwdm1* was indispensable for the wax crystals formation and its mutation resulted in glossy appearance in Chinese cabbage.

## Introduction

Cuticle wax is a waterproof layer covering the plant’s surface (Lewandowska et al., 2020), which mainly controls non-stomatal water loss. Cuticle wax could be altered by various abiotic or biotic factors (Kosma et al., 2009). Plants grown in drought conditions normally deposit more wax (Kunst and Samuels, 2003). In addition, the waterproof nature of cuticle wax is conducive to removing dust, bacteria, and fungus from plant surfaces (Yang et al., 2018). Leafy vegetables with epicuticular wax crystal deficiency are usually glossy and tender and tend to have higher commercial value. Previous studies have reported glossy mutants in leafy vegetables, such as cabbage, cai-tai, non-heading Chinese cabbage and Chinese cabbage (Ji et al. 2018; Liu et al. 2017b; Liu et al. 2017c; Wang et al. 2019; Wang et al. 2017; Yang et al. 2022a; Yang et al. 2022b; Zhang et al. 2013). Therefore, breeding for the epicuticular wax crystal deficiency products is an approach to improving the commercial quality of leaf vegetables (Ji et al., 2018; Liu et al., 2021a).

The composition of cuticle wax is complex, generally including very-long-chain fatty acids (VLCFAs) and their derivatives (Li et al., 2008). The composition of cuticle wax varies widely across diverse species and environments (Yeats and Rose, 2013). VLCFAs synthesized in epidermal cells are precursors for wax synthesis, which is divided into two stages and carried out in different cellular compartments. In the first stage, in the plastid, the *de novo* fatty acid synthesis under the action of fatty acid synthase complex (FAS) generates C16 and C18 acyl chains (Kunst and Samuels, 2003; Lewandowska et al., 2020). In the second stage, C16 and C18 fatty acids generate VLCFAs by the act of the fatty acid elongases (FAE) which are composed of four enzymes *β*-ketoacyl-CoA synthase (KCS) (James et al., 1995), *β*-ketoacyl-CoA reductase (KCR) (Xu et al., 1997), *β*-hydroxyacyl-CoA dehydratase (HCD), and enoyl-CoA reductase (ECR) (Yeats and Rose, 2013). There are two pathways of VLCFAs modification. One is the alkane-forming pathway, which produces aldehydes, alkanes, secondary alcohols, and ketones, and the other is the alcohol-forming pathway, which is responsible for the synthesis of primary alcohols and esters (Jetter and Kunst, 2008).

In *Arabidopsis*, many key enzyme genes involed in the VLCFAs modification were cloned and functionally verified. *CER1*, *CER3*, and *MAH1* have been identified as key genes in the alkane-forming pathway. The *CER1* gene encodes an aldehyde decarboxylase converting fatty acids into alkanes (Bourdenx et al., 2011). Expression of *CER1* is induced by osmotic and ABA stress, both of which can rapidly regulate the alkane biosynthesis to accumulate more wax (Bourdenx et al., 2011). Similar results were obtained in wheat (Li et al., 2019), apple (Qi et al., 2018), and rice (Yu et al., 2008). The amino acid sequences between CER1 and CER3 proteins shared a high consistency. Strict co-expression of *CER1* and *CER3* has been reported to synthesize VLC alkane synthesis in yeast strains (Bernard et al., 2012). *MAH1* encodes a midchain alkane hydroxylase and oxidizes VLCFAs to secondary alcohols, which in turn generates ketones (Greer et al., 2007). Whereas, the wax component produced from the alcohol-forming pathway in *Arabidopsis* leaves only accounts for approximately 20% of the total wax (Jetter and Kunst, 2008). Consequently, enzyme genes encoding relevant enzymes in alcohol synthesis pathways are less than those in alkane synthesis pathways. *WSD1* and *CER4* have been reported to participate in the alcohol synthesis pathway. *WSD1* is a bifunctional enzyme whose main activity is wax ester synthase (WS) activity, acting on the alcohol-forming pathway (Abdullah et al., 2021). *CER4* encodes an alcohol-forming fatty acyl-CoA reductase, responsible for primary alcohol and alkyl ester synthesis (Doan et al., 2009). The AtCER4 protein can promote the accumulation of C24:0 and C26:0 primary alcohols, which has been verified in yeast (*Saccharomyces cerevisiae*). Subcellular localization showed that the AtCER4 protein was localized to the endoplasmic reticulum. The result of qRT-PCR figured that *AtCER4* was expressed in flower, stem, leaf, root, and silique (Rowland et al., 2006).

Although many wax biosynthetic genes have been cloned and their functions had been verified in Arabidopsis, few genes have been characterized in Chinese cabbage. In this study, we used a pair of allelic epicuticular wax crystal deficiency mutant *wdm1* and *wdm7* as materials to clone the mutant gene in Chinese cabbage. We observed the cuticle wax morphology by Cryo-scanning electron microscopy (Cryo-SEM), determined the composition of wax by GC-MS, and analyzed the genetic characteristics of the epicuticular wax crystal deficiency trait. We found the candidate mutant gene by MutMap and validated it by KASP. Finally, we verified its function by allelic variation.

## Materials and methods

### Plant materials

‘FT’ is a Chinese cabbage DH line and derives from the variety Fukuda 50. The germinated seeds of ‘FT’ were treated with 0.8% EMS (Gao et al., 2022), and the epicuticular wax crystal deficiency mutant was screened in its M2. A total of 8 mutants with stably inherited epicuticular wax crystal deficiency were identified and named as *wdm1, wdm2, wdm3, wdm4, wdm5, wdm6, wdm7*, and *wdm8*. Compared with ‘FT’, the epicuticular wax crystal deficiency mutant exhibited less epidermal wax on the whole plant, which was more obvious at the bolting and flowering stages. Other than that, there are no other phenotypical differences between mutant and wild type. Among these mutants, the *wdm1* and *wdm7* were selected as experimental materials. All plants were grown in a greenhouse at Shenyang Agriculture University. In late August, the seeds were placed on infiltrated filter paper until germination and then were transferred to a 4-degree refrigerator for 15-day vernalization. Subsequently, the vernalized seeds were sowed in the plug and grew in a greenhouse. After 25 days, the seedlings were transplanted into the nutrition bowl in a greenhouse.

### Cryo-SEM analysis and GC–MS analysis

Cryo-scanning electron microscopy was used to observe the epidermal wax morphology of ‘FT’ and mutants. Fresh leaves were cut into 1 mm × 3 mm strips and fastened to the sample disk. The sample disk was first loaded into the transmission device, and then immersed in liquid nitrogen for precooling. The transmission device was installed in the preparation chamber, and the sample disk was transferred to the preparation chamber after the vacuum is pumped. Sublimate the water in the sample and spray the sample with gold before observing (Liu et al., 2021a).

Equal-area leaves of ‘FT’ and mutant was cut and applied in the wax analysis. The wax extraction method was as previously reported (Liu et al., 2021a). The composition of the sample was determined by AGILENT 6890-5973N installed with TG-5ms (30 m*250 um*0.25 μm). The GC program utilized was on the strength of previous reports (Wang et al., 2017).

### Genetic analysis and allele test

Eight mutants were separately crossed with ‘FT’ to construct F_1_, F_2_, and BC_1_ progenies to carry out the genetic analysis. F_2_ and BC_1_ segregation ratios of trait were analyzed by the Chi-square test. Mutant gene function could be verified by allelic mutants (Fu et al., 2020; Zhao et al., 2021). To verify whether there were allelic mutations, we crossed the eight materials with each other. All plants were grown in a greenhouse at Shenyang Agricultural University.

### MutMap and data analysis

We employed a modified MutMap method to identify the candidate gene(Gao et al., 2020). Fifty epicuticular wax crystal deficiency mutant plants from F_2_ were chosen for DNA extraction. Equal amounts of DNA were extracted from each sample and mixed to form an epicuticular wax crystal deficiency mutant pool (HC-pool). DNA from the two parental plants and the HC-pool were re-sequenced by adopting a NovaSeq 6000 System sequencer (Illumina, San Diego, USA). Introducing Burrows-Wheeler Aligner (BWA), sequencing results were aligned to the Brassica reference genome sequence (http://brassicadb.cn/#/Download_genome/Brassica_Genome_data/Brara_Chiifu_V3.0). GATK was used to detect SNPs and INDELs (Liu et al., 2021a). Gene function annotations referred to *Brassica* Database (BRAD) (Wang et al., 2020).

### SNP genotyping by KASP

Kompetitive allele-specific PCR (KASP) was used to detect whether the SNPs identified by MutMap were co-segregated with epicuticular wax crystal deficiency traits and to recognize candidate genes. The DNA samples of 89 F_2_ glossy individuals from *wdm1*, 3 F_1_ individuals, 2 ‘FT’ individuals and 2 *wdm1* were prepared for genotyping analysis. The DNA samples of 90 glossy individuals from F_2_ of *wdm7*, 2 F_1_ individuals, 2 ‘FT’ individuals and 2 *wdm7* were used for genotyping analysis.

### Cloning candidate gene in *wdm1* and *wdm7*

The full length of candidate genes were amplified by full-lengths PCR primers in ‘FT’, *wdm1*, and *wdm7*. After the purification of PCR products with a Gel Extraction Kit (CWBIO, Beijing, China), we introduced the purified PCR products into a pGEM-T Easy Vector (Promega, USA). Then, the recombinant vector was transformed into the Top 10 competent cells (CWBIO, Beijing, China). The positive clones were sequenced by GENEWIZ (Tianjin, China).

### Bioinformatic analysis of Brwdm1

The conserved domain of Brwdm1, sequenced from the *Brassica* database, was analyzed online at GSDS2.0 (http://gsds.gao-lab.org/), as well as SMART (https://smart.embl-heidelberg.de/). 50 amino acid sequences of Brwdm1 were put to use in constructing the three-dimensional structure through SWISS-MODEL (https://swissmodel.expasy.org/). The amino acid sequence of Brwdm1 homologs was acquired from *Brassica* database BLAST. We brought it to DNAMAN V6 (Lynnon BioSoft, Canada) for sequence alignment.

### RNA isolation and relative expression analysis

The expression of candidate genes in the root, stem, leaf, flower bud and silique parts were detected by quantitative real-time PCR (qRT-PCR). Using an RNA extraction Kit (TIANGEN, Beijing, China), total RNA was extracted from *wdm1* and ‘FT’. FastQuant RT Super Mix (TIANGEN) was used to synthesize first-strand cDNA. Specific primers design and qRT-PCR were performed as previously reported (Liu et al., 2021a).

## Results

### Characteristics of epicuticular wax crystal deficiency mutants

**Figure 1:**
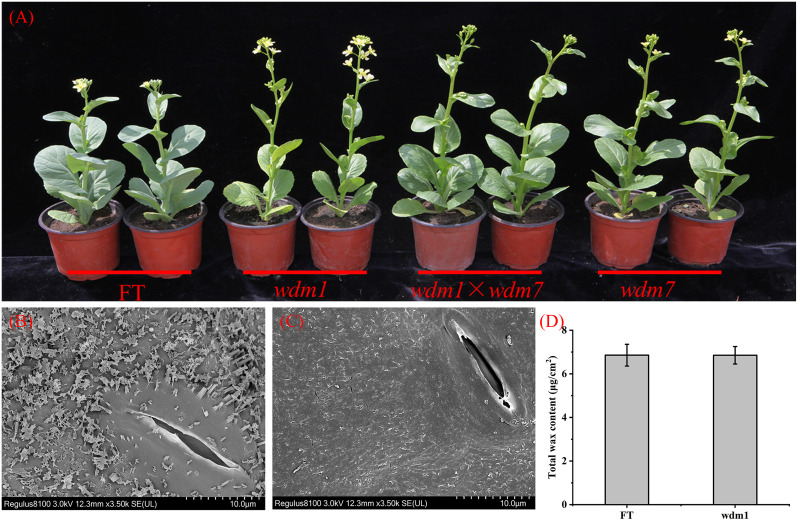
Phenotypic characterization of the ‘FT’ and mutants. **(A)** All the aerial organs of ‘FT’ were glaucous, while mutants displayed epicuticular wax crystal deficiency. The hybrid progenies from *wdm1* and *wdm7* appeared epicuticular wax crystal deficiency. SEM images of **(B)** ‘FT’ leaf and **(C)** *wdm1* leaf. The *wdm1* had less epidermal wax. **(D)** Total cuticle wax coverage and Wax coverage is expressed as μg/cm^–2^ leaf surface area. Values are means ± SE (n = 3).

WT (‘FT’) is a Chinese cabbage (*Brassica campestris* L. ssp. *pekinensis*) DH line with a whitish appearance. The epicuticular wax crystal deficiency mutants were obtained in an EMS-mutagenized population of ‘FT’. During the bolting and flowering stage, all the aerial organs, including siliques, buds, stems, and leaves were glaucous, while mutants displayed glossy phenotype in the corresponding organs. Except for the wax trait, there were no obvious differences between mutant and ‘FT’ (**Figure 1A**).

### The epicuticular wax crystal deficiency character was controlled by a recessive nuclear gene

‘FT’ was crossed with *wdm1* to generate F_1_. All the F_1_ plants demonstrated the whitish phenotype consistent with ‘FT’. Among 800 F_2_ individuals, 594 plants were whitish and 206 plants were epicuticular wax crystal deficiency phenotypes. The segregation ratio was close to 3:1 predicted by the Chi-square test (χ^2 =^ 0.24 < ${\text{χ}}^{2}{\;}_{0.05}$ = 3.841). In addition, the segregation ratios of BC_1_P_2_ (F_1_×*wdm1*) were close to 1:1 (χ^2 =^ 0.38). These results exhibited that the epicuticular wax crystal deficiency character of *wdm1* was regulated by a single recessive nuclear gene. The identical approach was employed to analyze the genetic characteristics of the remaining seven mutants, and the results manifested that their epicuticular wax crystal deficiency character was severally controlled by a recessive nuclear gene (**Table 1**).

**Table 1:** Genetic analysis of eight epicuticular wax crystal deficiency mutants.

Mutant	F_1_ Wax/Wax deficiency	F_2_ Wax/Wax deficiency	χ2 ( ${\text{χ}}^{2}{\;}_{0.05}$ =3.841)	BC_1_P_1_ Wax/Wax deficiency	BC_1_P_2_ Wax/Wax deficiency	χ2 ( ${\text{χ}}^{2}{\;}_{0.05}$ =3.841)
*wdm1*	50/0	594/206	0.24	100/0	112/103	0.38
*wdm2*	50/0	532/168	0.37	100/0	92/110	0.8
*wdm3*	50/0	433/130	1.09	120/0	105/118	0.38
*wdm4*	50/0	569/181	0.3	100/0	125/112	0.71
*wdm5*	50/0	587/180	0.94	150/0	89/81	0.18
*wdm6*	50/0	410/145	0.41	100/0	78/66	0.5
*wdm7*	50/0	392/108	3.08	150/0	95/85	0.56
*wdm8*	50/0	580/188	0.11	100/0	122/108	0.19

BC_1_P_1_: F_1_×FT. BC_1_P_2_: F_1_×*wdm*.

### *wdm1* and *wdm7* were allelic epicuticular wax crystal deficiency mutants

The genetic characterization of these eight mutants was recessive inheritance. After crossing *wdm1* and *wdm7*, we found that all the progenies were epicuticular wax crystal deficiency phenotypes (**Figure 1A**), which suggested that the epicuticular wax crystal deficiency phenotype of *wdm1* and *wdm7* was controlled by an identical gene. Similarly, three other groups of allelic mutants: *wdm2* and *wdm5; wdm3* and *wdm6*; *wdm4* and *wdm8*, were identified (**Table 2**).

**Table 2:** Allele test of eight epicuticular wax crystal deficiency mutants.

	*wdm1*	*wdm2*	*wdm3*	*wdm4*	*wdm5*	*wdm6*	*wdm7*	*wdm8*
*wdm1*	–							
*wdm2*	+	–						
*wdm3*	+	+	–					
*wdm4*	+	+	+	–				
*wdm5*	+	–	+	+	–			
*wdm6*	+	+	–	+	+	–		
*wdm7*	–	+	+	+	+	+	–	
*wdm8*	+	+	+	–	+	+	+	–

The positive sign “+” indicates wax. The negative sign“-” indicates epicuticular wax crystal deficiency.

### *wdm1* and *wdm7* had less epidermal wax crystals

The Cryo-SEM result revealed that flake-and-rod-shaped wax crystals continuously and densely covered the surface of the ‘FT’ leaf, while the filamentous waxy crystals were sparsely distributed on the surface of the *wdm1* leaf (**Figures 1B, C**).

### *wdm1* decreased in leave primary alcohol and ester

**Figure 2:**
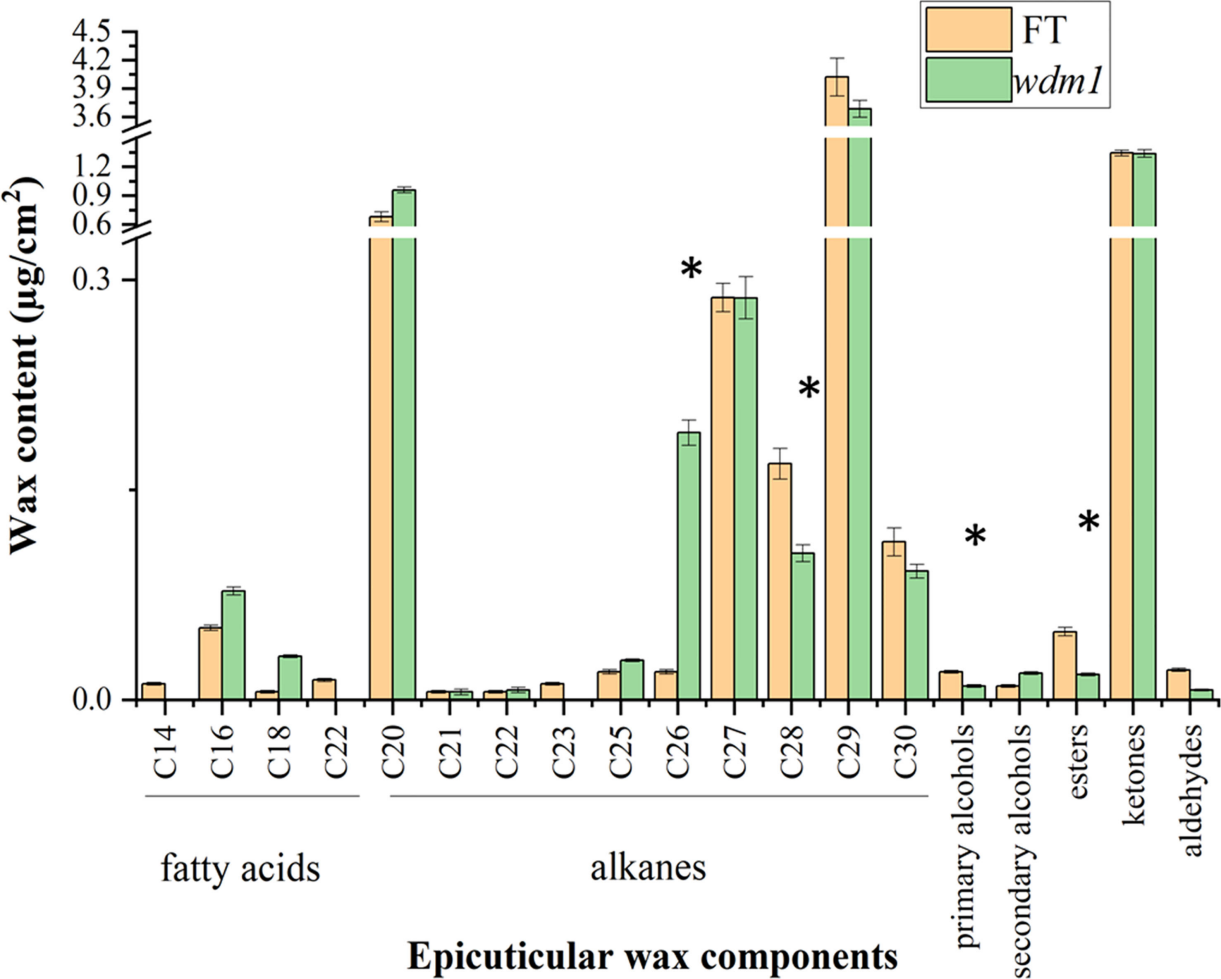
Epicuticle wax components of ‘FT’ and *wdm1* leaves. Mutant *wdm1* decreased in leave primary alcohol and ester, but elevated secondary alcohol content compared to ‘FT’. Asterisks indicate statistically significant differences determined using a t-test (*P < 0.05).

We measured the wax content and composition of ‘FT’ and *wdm1* by GC-MS, and surprisingly, the total amount of wax in the leaf epidermis of ‘FT’ and *wdm1* was almost the same (**Figure 1D**). Compared with ‘FT’, the primary alcohol and ester content in *wdm1* decreased by 50.5% and 62.4%, respectively, while the secondary alcohol content doubled. In addition, we noticed that the proportion of alkanes in the total wax of wild-type and mutant was large, reaching 77.8% and 78.1%. In particular, C28 alkanes decreased by 37.9% in *wdm1*, while C26 alkanes increased by 8.5 times (**Figure 2**).

### Preliminary mapping by MutMap

**Figure 3:**
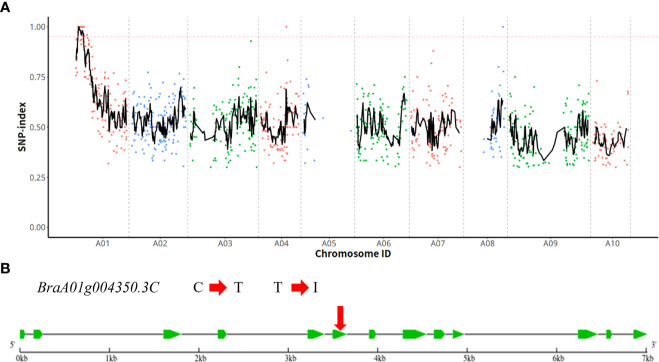
Identification of the candidate gene. **(A)** MutMap sliding window analysis. The x-axis and y-axis represent the positions of ten chromosomes and the SNP index, respectively. The dotted pink line is the index (0.95) threshold line. **(B)** Gene structure of *BraA01g004350.3C* and the location of non-synonymous SNPs in mutant *wdm1*.

After genome resequencing, we obtained 94,719,968, 54,123,946, and 140,604,782 high-quality reads for wild-type plant ‘FT’, *wdm1*, and HC pool, respectively. Thereinto, 98%, 96.89%, and 98.97% of high-quality reads from wild-type plant ‘FT’, *wdm1* and HC pool were aligned to the Chinese cabbage v. 3.0 reference genome, respectively. Through sequence alignment and mutation analysis software GATK, 1,847,414 SNPs were detected between ‘FT’ and *wdm1* pool. Assuming SNP index= 95% as the threshold, an 8.78 Mb region on chromosome A01 was identified as the candidate interval (**Figure 3A**). Only three SNPs (SNP 2,113,772, SNP 3,305,814, and SNP 4,248,438) were located in exons and caused non-synonymous mutations (**Table 3**).

**Table 3:** Three candidate SNPs information.

Gene ID	Pos	WT	Mut	SNP index	Exon ID	Annotation
*BraA01g004350.3C*	2113772	C	T	1	exon6	Fatty acyl-CoA reductase 3
*BraA01g006900.3C*	3305814	C	T	1	exon3	Myosin-binding protein 7
*BraA01g008650.3C*	4248438	G	A	1	exon4	Protein SPEAR3

### *BraA01g004350.3C* is the candidate gene

**Figure 4:**
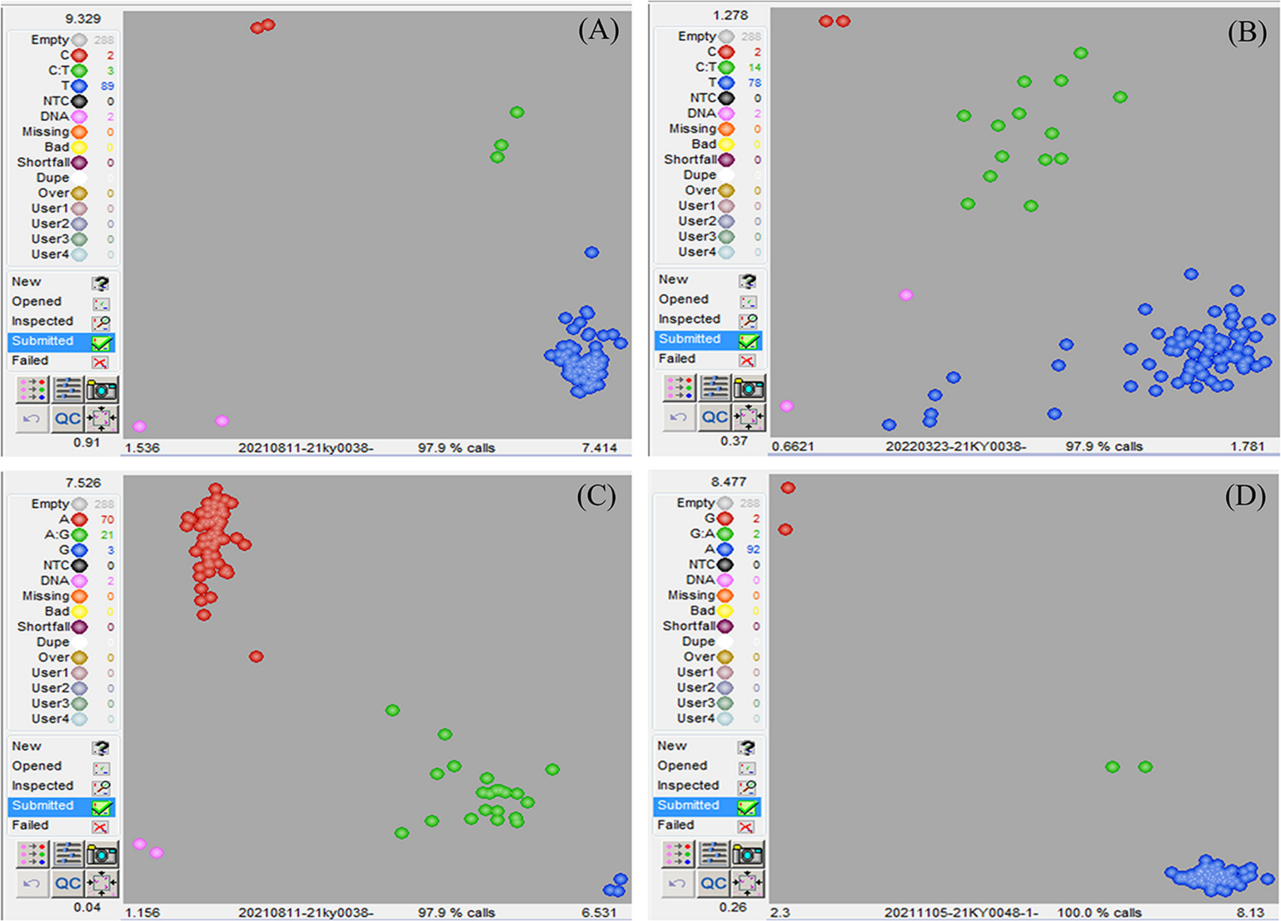
Identification of the candidate gene. 87 epicuticular wax crystal deficiency individuals from F_2_ of *wdm1*, 3 F_1_ individuals, 2 ‘FT’ individuals and 2 *wdm1* was used for **(A)** SNP 2,113,772, **(B)** SNP 3,305,814, and **(C)** SNP 4,248,438 genotyping analysis. **(A, B)** The blue dots, red dots and green dots severally represented T:T genotype, C: C and C: T genotype. **(C)** The blue dots, red dots and green dots separately stood for G: G genotype, A:A and G: A genotype. The DNA samples of 90 glossy individuals from F_2_ of *wdm7*, 2 F_1_ individuals, 2 ‘FT’ individuals and 2 *wdm7* was used for **(D)** SNP 2,114,994 genotyping analysis.

In order to confirm the causal SNP, specific KASP genotyping primers were designed for three SNP mutations. The results depicted that SNP 2,113,772 in *wdm1* individuals had a T: T genotype, whereas F_1_ (‘FT’×*wdm1*) individuals and ‘FT’ individuals had a C: T and C: C genotype, respectively. The genotypic assay described that SNP 2,113,772 was the T: T genotype in the 87 F_2_ epicuticular wax crystal deficiency individuals (**Figure 4A**). By contrast, SNP 3,305,814 were explored as C: T or C: C genotypes and SNP 4,248,438 were detected as G: A genotypes in the 87 F_2_ epicuticular wax crystal deficiency individuals (**Figures 4B, C**). Therefore, we considered that the SNP 2,113,772 of *BraA01g004350.3C* co-segregated with the epicuticular wax crystal deficiency mutant phenotype. *BraA01g004350.3C* is homologous to Arabidopsis *AtCER4* (*AT4G33790*) and encodes an alcohol-forming fatty acyl-CoA reductase (from the *Brassica* database). Consequently, we hypothesized that *BraA01g004350.3C (Brwdm1)* was the candidate causal gene.

### Mutation of *Brwdm1* leads to the epicuticular wax crystal deficiency

**Figure 5:**
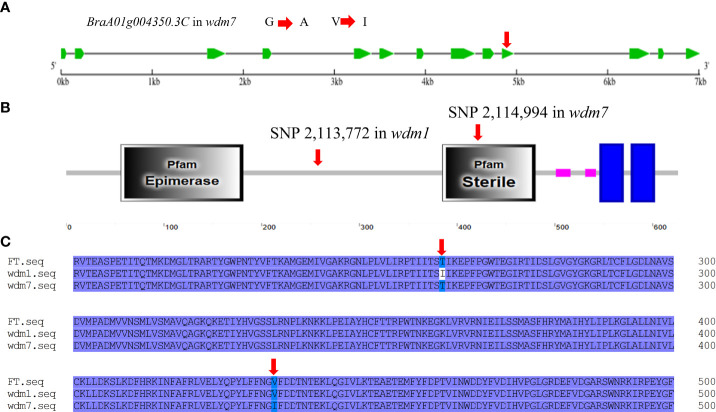
The mutantion of *Brwdm1* in *wdm7.* **(A)** Gene structure of *Brwdm1* and the location of non-synonymous SNP 2,114,994 in *wdm7*. **(B)** The conserved domain of Brwdm1 as well as the location of SNP 2,113,772 and SNP 2,114,994. Grey boxes were conserved domains. **(C)** Partial alignment of protein sequences of Brwdm1 of ‘FT,’ *wdm1* and *wdm7. wdm1* (the 262nd: T to I). *wdm7* (the 434th: V to I).

Learn from the *Brassica* database, *Brwdm1* was 7015 bp in length and consisted of 13 exons. We cloned the full length of *Brwdm1* from ‘FT’, *wdm1*, and *wdm7* (**Figure S1**). Only the SNP 2,113,772 (C to T) was found for *Brwdm1* in *wdm1* causing the amino acid change from threonine (T) into isoleucine (I), which was consistent with the MutMap sequencing report (**Figure 3B**; **Figure 4B**). Correspondingly, we discovered that there was a SNP (G to A), named SNP 2,114,994, in the 10^th^ exon of *Brwdm1* in *wdm7*, which led to the change of amino acid from valine (V) into isoleucine (I) (**Figure 5A**). The genotyping results of SNP 2,114,994 showed that *wdm7* had a A:A genotype, whereas F_1_ and ‘FT’ had a G:A and G:G genotype, respectively. And SNP 2,114,994 was the A:A genotype in the 90 F_2_ glossy individuals (**Figure 4D**). This verified that the mutation of the gene *Brwdm1* lead to the glossy phenotype.

### Structural and phylogenetic analyses of Brwdm1

**Figure 6:**
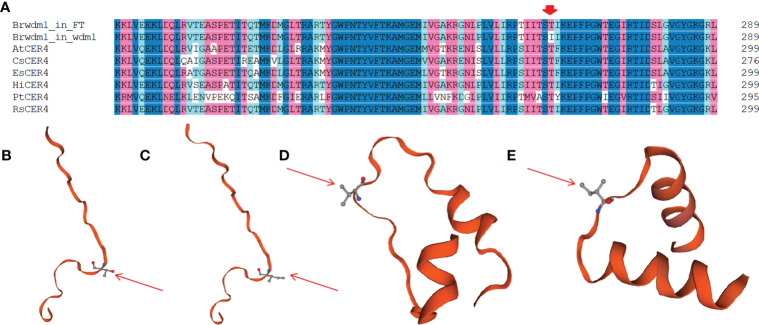
Gene sequence alignment and predicted three-dimensional structure of Brwdm1 protein. **(A)** Partial aligning to the amino acid sequences of Brwdm1 and these homologues. The CER4 protein accession numbers are as follows: *Arabidopsis thaliana*, AtCER4 (NP_567936.5); *Camelina sativa*, CsCER4 (AIE57506.1); *Populus tomentosa*, PtCER4 (AEV53412.1); *Raphanus sativus*, RsCER4 (XP_018478019.1); *Eutrema salsugineum*, EsCER4 (XP_006412311.1) and *Hirschfeldia incana*, HiCER4 (KAJ0252035.1). Partial three-dimensional structure of BrCER4 protein in ‘FT’ **(B)** and *wdm1* **(C)** Red arrows: amino acid residue 262 (Thr) of ‘FT’ **(B)** and (Ile) of *wdm1* **(C)**. Partial three-dimensional structure of Brwdm1 protein in ‘FT’ **(D)** and *wdm7* ***(*E*)*** Red arrows: amino acid residue 434 (Val) of ‘FT’ **(D)** and (Ile) of *wdm7* **(E)**.

Brwdm1 protein contains an epimerase domain (56-181), a sterile domain (385-480), and two transmembrane helix regions (547-569 and 579-601). This sterile domain represents the C-terminal domain of fatty acyl CoA reductases, which are involved in cuticle wax biosynthesis. The amino acid transformations in *wdm7* (the 434^th^: V to I) occurred in the sterile domain, while those of *wdm1* (the 262^nd^: T to I) were not (**Figures 5B, C**). We found several homologs of Brwdm1 from NCBI by BLAST. The amino acid sequences were aligned to understand the relationship between Brwdm1 and these homologs. The results demonstrated that the 262^nd^ amino acid was conservative (**Figure 6A**). Nucleotide substitutions at both 262^nd^ and 434^th^ issued changes in the three-dimensional structure of the Brwdm1 protein (**Figures 6B–E**). Therefore, we inferred that the protein conformational changes caused by amino acid substitutions made for the loss of Brwdm1 function, which in turn led to the epicuticular wax crystal deficiency phenotype.

### Relative expression analysis of candidate genes

**Figure 7:**
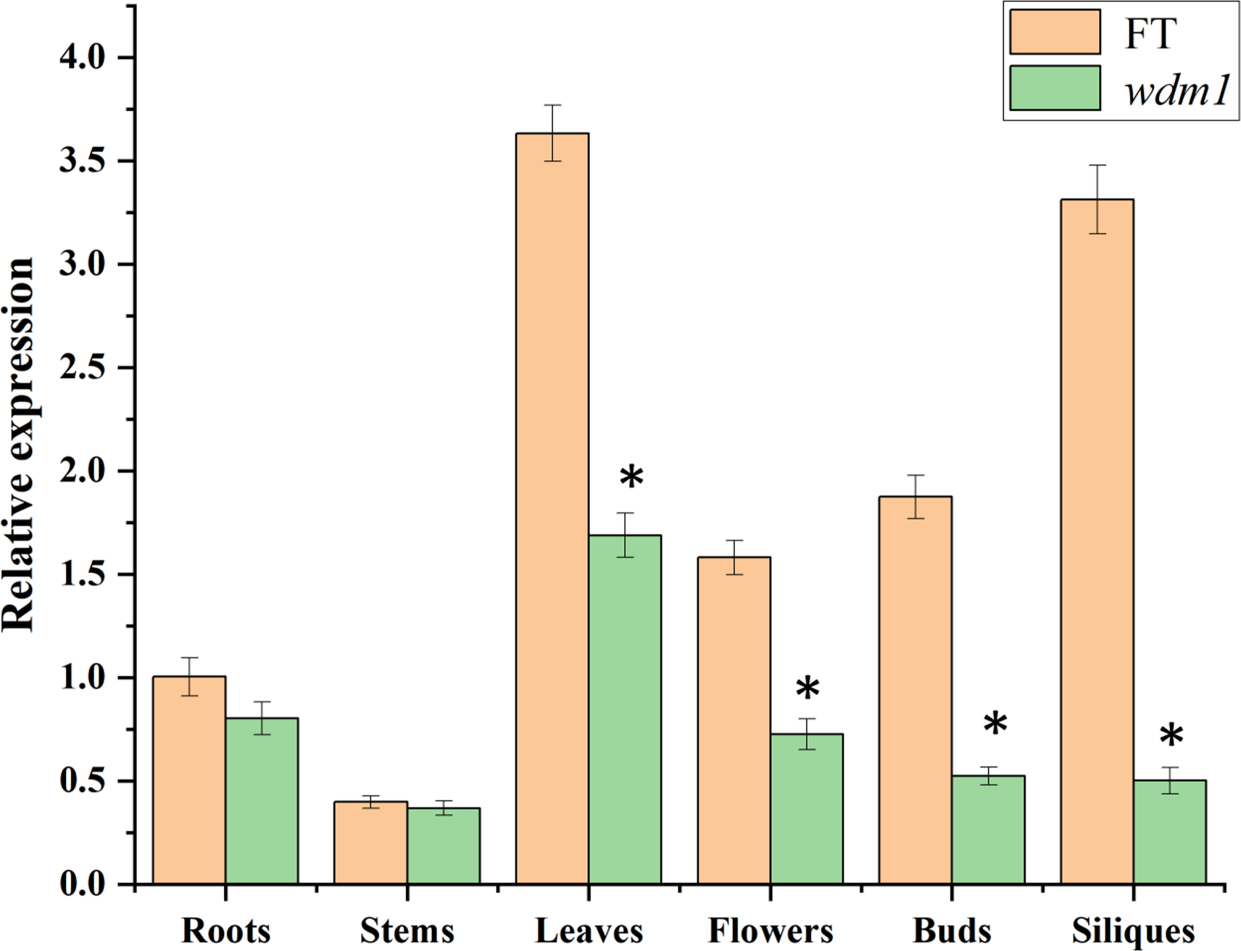
Relative expression levels of *Brwdm1* in different organs of ‘FT’ and *wdm1*. Asterisks indicate statistically significant differences determined using a t-test (*P < 0.05).

The relative expression of *Brwdm1* of the various organs from ‘FT’ and *wdm1* was determined by qRT-PCR. The results showed that the expression levels of *Brwdm1* in the leaves of FT and *wdm1* was the highest, respectively. Compared with the wild type, the relative expression of *Brwdm1* in the leaves, flowers, buds and siliques of *wdm1* was significantly decreased (**Figure 7**).

## Discussion

Plant mutants acquired by artificial mutagenesis possess widely and randomly mutated traits, which are ideal materials for germplasm innovation. As an excellent germplasm resource and its potential breeding value for improved quality, epicuticular wax crystal deficiency mutants have been reported in a variety of plants, such as navel orange, cabbage, cucumber, tomato, and apple (Liu et al., 2012; Wang et al., 2015; Liu et al., 2017c; Zhang et al., 2019; Xiong et al., 2020). The mutants in our experiment were derived from an EMS mutagenesis population. Since the wild-type ‘FT’ was a DH line, our mutants were excellent material for investigating gene functions. In this experiment, eight epicuticular wax crystal deficiency mutants were identified, and their epicuticular wax crystal deficiency traits followed recessive inheritance. For a class of mutants with similar traits and genetic characteristics, the allelism test could be employed to determine whether they were controlled by an identical mutant gene. Among the eight mutants, we recognized four groups of allelic mutants by the allelism test, and one group of them (*wdm1* and *wdm7*) was applied as experimental material to clone the epicuticular wax crystal deficiency mutant gene. Mutmap and KASP tests proved that *BraA01g004350.3C (Brwdm1)* was the target gene.

Currently, limitations still existed in the implementation of the transgenic technology in Chinese cabbage, while allelic mutant analysis could replace the transgenic technology to verify the gene functions. Allelic mutants have been applied in some crops for gene function verification, such as in lettuce (Huo et al., 2016), sorghum (Jiao et al., 2017), and cucumber (Zhang et al., 2018). Through BSR-Seq and whole-genome re-sequencing, gene *BrDVR* was identified as a candidate gene contributing to whole-plant pale green in Chinese cabbage mutant *pem1*. In *pem2*, which was an allelic mutant of *pem1*, a SNP was identified in *BrDVR* (Zhao et al., 2021). Herein, we confirmed *Brwdm1* was the candidate for epicuticular wax crystal deficiency in *wdm1* and found another SNP occurred in *Brwdm1* from its allelic mutant *wdm7*.

*Brwdm1* encodes an alcohol-forming fatty acyl-CoA reductase (FAR), involved in cuticle wax biosynthesis. The specific expression of apple *CER4* homolog in the fruit epidermis resulted in the abundance of C30, C28 and C26 primary alcohols in the fruit wax of cv Florina (Albert et al., 2012). In navel orange, the reduced expression of *CsCER4-LIKE1* and *CsCER4-LIKE3* led to a decrease in primary alcohol content in the waxy fruit epidermis (Liu et al., 2015). The *CsCER4* gene was cloned in cucumber, and its expression level was significantly diverse between lines 3413 (glossy type) and 3401 (waxy type) (Wang et al., 2018). The substrates of alcohol-forming pathway and the alkane-forming pathway are both very-long-chain fatty acids (VLCFAs). In *Arabidopsis cer4* mutants, the content of primary alcohols and wax esters was significantly decreased, and the content of aldehydes, alkanes, secondary alcohols, and ketones was slightly elevated (Rowland et al., 2006). In *Arabidopsis cer1* mutants, the content of aldehydes, alkanes, secondary alcohol and ketones, as well as primary alcohols and esters was significantly decreased (Bourdenx et al., 2011). There is a mutual interaction between the two forming pathways. Therefore, mutations in *BrCER4* also affected alkane content in Chinese cabbage. Wax crystals and wax films are two forms of wax accumulation in the epidermis. Wax crystals give the plant a glaucous appearance and wax films give the plant a glossy appearance. In our experiment, all the aerial organs of *wdm1* and *wdm7* were glossy and less wax crystals were observed on the surface of the *wdm1* leaf than ‘FT’ under Cryo-scanning electron microscope. The total wax content in our experiment includes not only wax crystals content, but also wax films content. These results were similar to those reported in *Arabidopsis* (Rowland et al., 2006), indicating that *CER4* plays a conserved role across multiple species. Nevertheless, it remains to be explored whether the acyl reduction product is fundamental for the formation of wax crystals.

The homologous genes of *AtCER4* have been cloned in *Brassica napus*, cabbage, and purple cai-tai, and they were reported to be involved in the wax biosynthesis pathway. In *Brassica napus*, a 157-bp DNA deletion in *BnA1.CER4* and a 1905-bp DNA insertion in *BnC1.CER4* were confirmed to generate wax deficiency in eceriferum mutant *Nilla Glossy*, which was controlled by these two recessive alleles (Liu et al., 2021b). In cabbage, a SNP in *Bol013612* altered the splice site, giving rise to a six-nucleotide insertion into the cDNA. And this mutation interfered with gene function, which brought about a glossy appearance in mutant LD10G (Liu et al., 2017a). In purple cai-ta, a 39-bp deletion of *Bra011470* was responsible for the glossy phenotype (Wang et al., 2019). By aligning Brwdm1 with its homologous proteins, we discovered that the mutant site of Brwdm1 in *wdm1* was conserved, and the nucleotide substitution at 262^nd^ changed the three-dimensional structure.

The epicuticular wax crystal deficiency mutants identified in this experiment had the glossy appearance, tender taste, and similar growth potential to the wild-type ‘FT’, and provided an excellent gene resource for commercial quality breeding in Chinese cabbage. Through back-crossing breeding, the glossy and tender characteristics could be transferred into other excellent lines to cultivate high-quality varieties.

## Data availability statement

The original contributions presented in the study are publicly available. This data can be found here: https://www.ncbi.nlm.nih.gov/accession: PRJNA941501.

## Author contributions

HF and GS designed the experiments. GS performed the experiments and wrote the manuscript. CL and JR assisted in the screening of mutants. HF and BF revised the manuscript. All authors contributed to the article and approved the submitted version.

## Supplementary material

Supplementary Figure 1 Cloning candidate gene *BraA01g004350.3C* in ‘FT’ and mutants. The full length of 7015 bp was cloned in four segments, which were 1769bp (F: 5’ GGCTTTTACCGATCACCATT 3’; R: 5’ ACGTCCCCATTGACAACAGT 3’), 1802bp (F: 5’ GGGAAGGACTTGTACAGGGTA 3’; R: 5’ GAACCTGGTGAGTCCCATATC 3’), 1655bp (F: 5’ CTCCTGAAACCATCACTCAAA 3’; 5’ AAACAAAGGTTACACAAATGACAT 3’), and 2210bp (F: 5’ AAGACAGAAGCCGAAACCGA 3’; 5’ TTCGGAGAACCTTAGCCCAT 3’), respectively. When designing the primers, we set overlaps between adjacent segments to ensure that the segments can be properly spliced after sequencing. The first 1769bp segment contains part of the promoter sequence, and the last 2210bp segment contains part of the downstream sequence of the gene. After sequencing, the four segments can be spliced together and contain a full length of *BraA01g004350.3C* (7015 bp).
