# Supplementary figures and images for: Identification of an epicuticular wax crystal deficiency gene Brwdm1 in Chinese cabbage (Brassica campestris L. ssp. pekinensis)
Source: Front Plant Sci. 2023 May 30;14:1161181. doi: 10.3389/fpls.2023.1161181 (PMC10267742; doi:10.3389/fpls.2023.1161181)

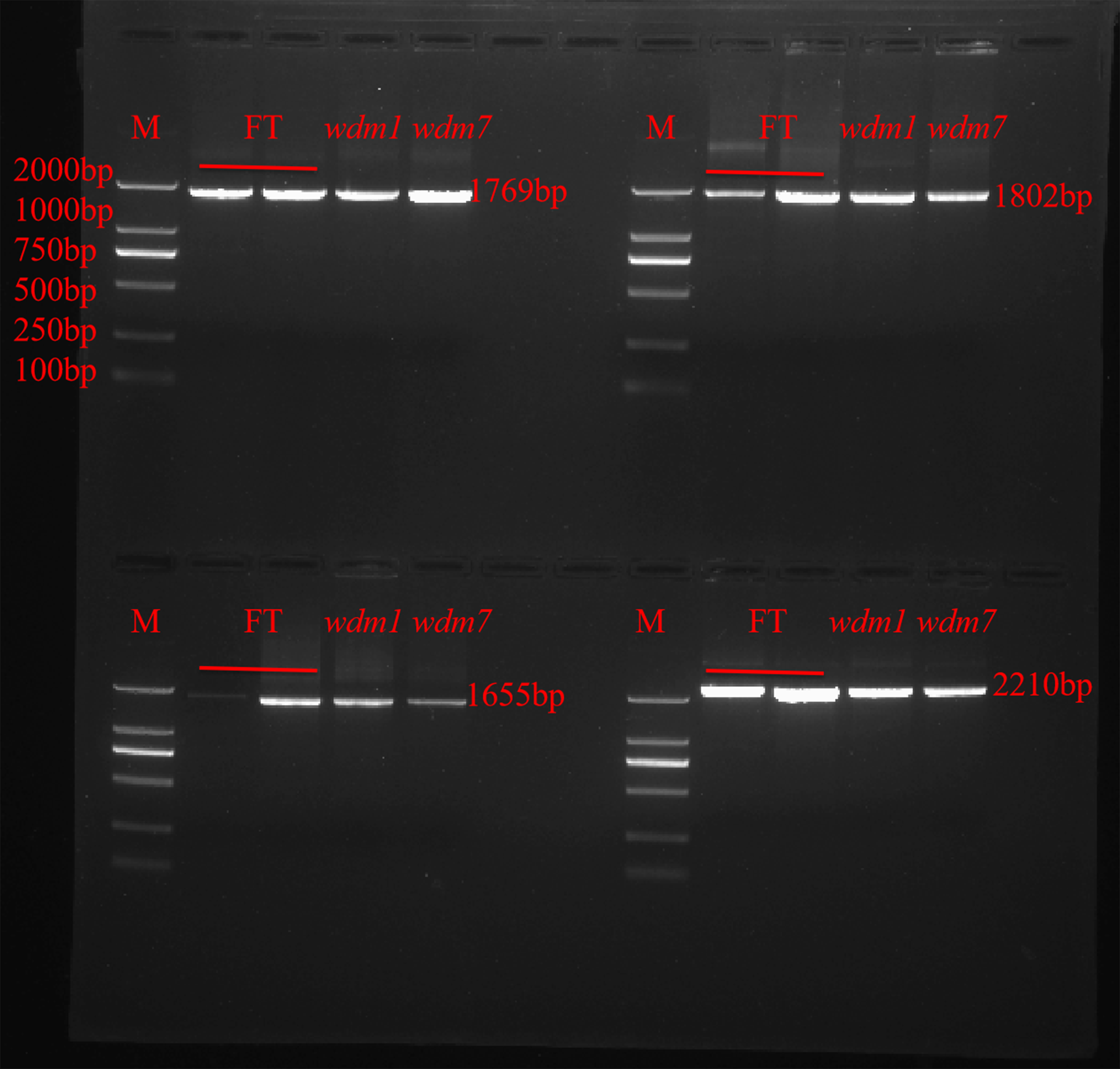

Supplement: Supplementary Figure 1 — Cloning candidate gene BraA01g004350.3C in ‘FT’ and mutants. The full length of 7015 bp was cloned in four segments, which were 1769bp (F: 5’ GGCTTTTACCGATCACCATT 3’; R: 5’ ACGTCCCCATTGACAACAGT 3’), 1802bp (F: 5’ GGGAAGGACTTGTACAGGGTA 3’; R: 5’ GAACCTGGTGAGTCCCATATC 3’), 1655bp (F: 5’ CTCCTGAAACCATCACTCAAA 3’; 5’ AAACAAAGGTTACACAAATGACAT 3’), and 2210bp (F: 5’ AAGACAGAAGCCGAAACCGA 3’; 5’ TTCGGAGAACCTTAGCCCAT 3’), respectively. When designing the primers, we set overlaps between adjacent segments to ensure that the segments can be properly spliced after sequencing. The first 1769bp segment contains part of the promoter sequence, and the last 2210bp segment contains part of the downstream sequence of the gene. After sequencing, the four segments can be spliced together and contain a full length of BraA01g004350.3C (7015 bp). [file Image_1.tiff]
